# Intramuscular iron supplementation enhances intestinal barrier function in weaned piglets challenged with enterotoxigenic *Escherichia coli*

**DOI:** 10.3389/fcimb.2025.1553639

**Published:** 2025-07-17

**Authors:** Chenying Tian, Xiaofeng Zhang, Yecheng Xu, Linfeng Miao, Jing Zhao, Qingqing Xiong, Yuhui Zhang, Shouchuan Jiang, Yu Han, Huahua Du

**Affiliations:** ^1^ Key Laboratory of Nutrition and Breeding for High-quality Animal Products of Zhejiang Province, College of Animal Sciences, Zhejiang University, Hangzhou, China; ^2^ Institute of Animal Husbandry and Veterinary Science, Zhejiang Academy of Agricultural Sciences, Hangzhou, China

**Keywords:** intramuscular iron supplementation, oral iron supplementation, weaned piglets, ETEC K88, diarrhea, intestinal barrier function

## Abstract

**Background:**

Since iron is an essential mineral for both host and microbial communities, how to scientifically replenish the iron in the context of bacterial infection has become a critical issue. The aim of this study was to compare the influence of intramuscular and oral iron supplementation on the progression of bacterial infection.

**Methods:**

Weaned piglets served as an experimental model for iron supplementation following enterotoxigenic *Escherichia coli* (ETEC) K88 infection. Piglets in control and oral iron supplementation groups received FeSO_4_ orally, while those in the intramuscular iron supplementation group were administered iron dextran (FeDex) via intramuscular injection. After challenge, piglets were euthanized, and serum and small intestinal tissues were collected for biochemical analysis, histological examination, inflammatory response assessment, gut microbiota profiling, and iron metabolism evaluation.

**Results:**

Intramuscular iron supplementation alleviated the clinical symptoms of bacterial infection, decreasing the diarrhea rate by 53% and mitigating the inflammatory response with lower serum levels of pro-inflammatory cytokines, such as TNF-α, IL-6, and IL-8. Compared to oral iron supplementation, intramuscular iron supplementation significantly mitigated the intestinal damage caused by ETEC K88 infection by increasing the ratio of villus length to crypt depth, and repairing epithelial tight junction. Furthermore, intramuscular iron supplementation also protected the function of intestinal goblet cells and improved iron metabolism of infected piglets.

**Conclusion:**

Intramuscular iron supplementation is more effective during infection than oral iron supplementation.

## Introduction

Iron is an essential trace element for maintaining the internal stability and normal physiological functions of animals, including hematopoiesis, oxygen transport, DNA synthesis, and energy metabolism (Ganz and Nemeth, 2015). During the first few weeks of life, piglets are prone to iron deficiency anemia due to low iron storage, low iron content in sow milk, incomplete development of intestinal iron absorption mechanism and high iron demand for growth and development, resulting in severe iron metabolism disorders and various problems such as diarrhea, inflammation, and even death (Lachowicz et al., 2014). Intestinal infections like enterotoxigenic *Escherichia coli* (ETEC) K88 exacerbate iron dysregulation by degrading the tight junction proteins and simultaneously activating NF-κB-mediated pro-inflammatory pathways (Wang et al., 2024). By the time of weaning, piglets have exhausted their prenatal iron stores and iron obtained from neonatal supplements due to their rapid growth demands, necessitating appropriate artificial supplementation measures. Therefore, iron supplementation levels in commercial diets for post-weaning piglets are on par with or greater than the NRC’s (2012) guidelines (Yang et al., 2021). However, these highly iron enriched diets wouldn’t effectively improve iron homeostasis, and even increase the risk of infection sometimes because bacteria such as ETEC K88 exploit free iron for the synthesis of virulence factors (Fjelkner et al., 2024).

Post-weaning in piglets typically results in pronounced changes in intestinal morphology, characterized by damaged villi, which impairs the intestinal capacity for nutrient digestion and absorption, contributing to diarrhea (Agazzi et al., 2020). Furthermore, weaned piglets have increased intestinal mucosal permeability, delayed development of the mucosal barrier, and compromised mucosal integrity, all of which predispose them to bacterial invasion. ETEC K88 specifically targets intestinal epithelial cells via F4 fimbriae, disrupting barrier function and exacerbating intestinal permeability, a critical precursor to systemic inflammation (Moonens et al., 2015). The weaning stress also elicits significant alterations in the gut microbiota composition, reducing its stability and promoting the proliferation of harmful bacteria and toxin production, further exacerbating intestinal pathological changes. These issues are intensified in cases of intestinal infections, significantly reducing the piglets’ growth performance (Wang et al., 2021). Because bacteria also need iron for growth and reproduction, dietary iron supplementation may be counterproductive at this time. Our previous studies found that iron supplementation to infected mice would aggravate the infection of *Escherichia coli* O157:H7 and increase the mortality of infected mice (Yu et al., 2019), but intraperitoneal iron supplementation can alleviate intestinal inflammation in mice (Liang et al., 2021). Therefore, it is crucial to explore an appropriate way of iron supplementation to avoid exacerbating piglet diseases after weaning.

Emerging evidence suggests that iron supplementation strategies must balance systemic iron availability with intestinal mucosal immunity. For instance, intraperitoneal iron administration in mice reduced colonic inflammation by limiting pathogens’ access to luminal iron (Liang et al., 2021), while oral supplementation exacerbated ETEC O157:H7 virulence (Yu et al., 2019). Although many studies have explored the pros and cons of iron supplementation methods for iron-deficient piglets (Chen et al., 2019; Mazgaj et al., 2021), there is currently no specific research on the advantages and disadvantages of iron supplementation methods for bacterially infected pigs. Therefore, the aim of the present study was to assess the influence of intramuscular iron supplementation on intestinal morphology, inflammation, barrier function, and gut microbiota of weaned piglets challenged with ETEC K88, which is a typical pathogen casing diarrhea of piglets.

## Materials and methods

### Animal group and administrations

Every experimental procedure complied with current animal protection laws and was authorized by Zhejiang University’s Animal Care and Use Committee’s Guide for the Care and Use of Laboratory Animals. Twelve 28-day-old landrace × large white-binary weaned piglets were randomly divided into 3 groups: control group (CON+FeSO_4_), oral iron supplementation infected group (K88+FeSO_4_), and intramuscular iron supplementation infected group (K88+FeDex). The experimental period was 9 days with 3 days of prefeeding, and piglets were all fed a basal diet (20 mg Fe/kg) during 1-6 days. Infected piglets were challenged with ETEC K88 (50 mL 1×10^9^ CFU/mL per day) for 4-6 days. From 7-9 days, piglets in the CON+FeSO_4_ and K88+FeSO_4_ groups received FeSO_4_ orally (a total of 300 mg), whereas those in the K88+FeDex group were injected with iron dextran (a total of 300 mg). Piglets were slaughtered and sampled on day 10 of the experiment.

### Blood and serum parameters analysis

Hematological indices were determined using a Mindray automatic blood cell analyzer (Mindray, Shenzhen, China). Piglet serum was separated from blood by centrifugation at 3,000 g for 10 min. Serum iron assay kit and total iron binding capacity assay kit (Jiancheng Biology, Nanjing, China) were used to detect the serum iron content of piglets according to the manufacturer’s instructions. The unsaturated iron binding capacity was calculated by subtracting the value of total iron binding from the value of serum iron. Serum levels of immunoglobulin (Ig) A, IgG and IgM were analyzed by ELISA kits (Meibiao Biology, Jiangsu, China) using double antibody sandwich assay according to the manufacturer’s instructions. Absorbance values were determined with a Microplate Reader (Bio-Rad, 680, Hercules, CA, USA) at 450 nm.

### Histological analysis

Duodenum, jejunum, ileum and colon samples from piglets were placed in 4% paraformaldehyde overnight and embedded in paraffin. Embedded samples were cut into 5-μm thin sections. After deparaffinization, tissue sections were stained with hematoxylin for nuclei and eosin for cytoplasm. After staining, the tissue sections were dehydrated with graded concentrations of ethanol solutions and finally air dried. All sections were visualized using DM3000 microscope (Leica, Wetzlar, Germany).

### Transmission electron microscopy

Colonic tight junctions and microvilli structures were visualized using TEM. Animal intestines were cut into small tissue pieces and placed into 2.5% glutaraldehyde fixative 24 h at 4°C. The samples were fixed with 1% osmic acid solution for 1-2 h and treated with graded dehydration using different concentrations of ethanol (30%, 50%, 70%, and 80%). They were treated with 90% and 95% acetone solution for 15 min each, and placed in pure acetone for 20 min. The samples were taken into a mixture of acetone and Spurr resin (1:1 for 1 h and 1:3 for 3 h) and then in Spurr resin overnight. Tissues were placed in capsules, heated at 70°C for 9 h. Then stained with uranyl acetate and alkaline lead citrate for 15 min. The tissue sections were visualized using H-7650 TEM (Hitachi, Tokyo, Japan).

### Scanning electron microscope

The morphology of intestinal microvilli was observed by SEM. The tissue was placed in 2.5% glutaraldehyde solution and fixed for 24 h at 4°C. After fixation of the samples with 1% osmic acid solution for 1-2 h, they were extensively rinsed with phosphate buffer. Different gradient ethanol solutions were dehydrated for 15 min and then treated with 100% ethanol for another 20 min. Samples were transferred to a mixture of ethanol and isoamyl acetate (v: v = 1:1) for 30 min and then incubated with isoamyl acetate for 1 h. Drying was performed in a Hitachi HCP-2 critical point dryer. Finally, the dehydrated samples were coated with palladium and observed using a Philips SU8010 FASEM (Hitachi, Tokyo, Japan).

### Immunohistochemistry staining

Intestinal tissues were fixed in 4% paraformaldehyde at 4°C for 24 h. Endogenous peroxidase was blocked with 3% methanol hydrogen peroxide after dewaxing intestinal tissue sections. The sections were put into Tris-EDTA antigen retrieval solution, boiled for 5 min, and blocked with 3% bovine albumin for 1 h at room temperature. Tissue sections were incubated with anti-CD4^+^ antibody (1:500, Abcam, MA, USA) and anti-F4/80 antibody (1:5,000, Abcam, MA, USA) overnight at 4°C. Sections were stained with horseradish peroxidase for 30 min after 1 h incubation with secondary antibodies. The nuclei were finally stained with hematoxylin solution. All sections were observed by a DM3000 microscope (Leica, Wetzlar, Germany).

### PAS and AB-PAS staining

Periodic acid schiff (PAS) and alcian blue (AB)-PAS staining were used to identify goblet cells and the inner mucus layer of jejunal mucosa. Piglet’s jejunum was fixed in 4% paraformaldehyde solution for 24 h, embedded in paraffin and cut into 5-μm sections. Paraffin sections were then stained with PAS and AB. The number of goblet cells in sections was assessed by blindly counting the positive vacuoles. All colon sections were identified using a DM3000 microscope (Leica, Wetzlar, Germany).

### RNA extraction and RT-qPCR

Total RNA from intestines and livers were isolated using Trizol (Biosharp, Anhui, China). The RNA concentration was detected using Nanodrop 2000 spectrophotometer (Thermo Fisher Scientific, USA) and cDNA was synthesized using Hifair^®^ III 1st Strand cDNA Synthesis SuperMix for qPCR (Yeasen Biotechnology, Shanghai, China). Quantitative real-time PCR was carried out on ABI 7500 real-time PCR system (Thermo Fisher Scientific, USA) and using Hief UNICON^®^ qPCR SYBR Green Master Mix (Yeasen Biotechnology, China). The fold difference in mRNA expression was determined by 2^-ΔΔCt^ method and presented relative to endogenous β-actin mRNA. All reactions were verified at least in triplicate. The primer sequences are listed in **Table 1**.

**Table 1:** The primers used in quantitative real-time PCR.

Gene	Primer	Sequence 5′–3′
IL-6	Forward	TCCAGCATCATTGCATCATC
	Reverse	GGCTCCACTCACTCCACAAG
IL-8	Forward	TGAGAAGCAACAACAACAGCA
	Reverse	CAGCACAGGAATGAGGCATA
TNF-α	Forward	ACAGGCCAGCTCCCTCTTAT
	Reverse	CCTCGCCCTCCTGAATAAAT
iNOS	Forward	GGAGCCATCATGAACCCCAA
	Reverse	GTAGAAGCTCGTCTGGTGGG
IFN-γ	Forward	TTCAGCTTTGCGTGACTTTG
	Reverse	GGTCCACCATTAGGTACATCTG
IL-4	Forward	CCCGAGTGTCAAGTGGCTTA
	Reverse	TGATGATGCCGAAATAGCAG
IL-10	Forward	GCTGAAGACCCTCAGGCTGA
	Reverse	TTGCTCTTGTTTTCACAGGGC
ZO-1	Forward	TACCCTGCGGCTGGAAGA
	Reverse	GGACGGGACCTGCTCATAACT
Claudin-1	Forward	TCTTAGTTGCCACAGCATGG
	Reverse	CCAGTGAAGAGAGCCTGACC
Occludin	Forward	AGAGTCATAAGGTGGGGCAGT
	Reverse	CGCCCGTCGTGTAGTCTGTC
β-actin	Forward	GGATGCAGAAGGAGATCACG
	Reverse	ATCTGCTGGAAGGTGGACAG

### Gut microbiota analysis

Samples of cecal contents were collected and immediately frozen, and stored at -80°C. GENEWIZ was used to prepare next-generation sequencing libraries and perform Illumina MiSeq sequencing. The Magen Hipure Soil DNA kit was used to extract total genomic DNA from the samples. Amplicons were generated using 20-30 ng of DNA. The V3-V4 gene regions of the bacterial 16S rRNA gene were amplified with forward primer (338F-ACTCCTACGGGAGGCAGCAG) and reverse primer (806RGGACTACHVGGGTWTCTAAT). MiSeq platform was used to analyze bacterial communities, including operational taxonomic units (OTU) cluster analysis, species annotation, sample complexity analysis, comparison analysis of multiple samples, and significance analysis of community structure differences among populations.

### Sample size validation and statistical power analysis

Power analysis was performed using G*Power 3.1.97 software. *Post-hoc* power calculations employed ANOVA-based methodology at standard significance thresholds. Cohen’s f effect size metrics were assessed across all experimental endpoints, with established multiplicity corrections applied to address type I error inflation. This analytical approach validated the detection capacity of our experimental design for key barrier function parameters while characterizing statistical sensitivity for exploratory biomarkers. Monte Carlo simulation protocols further confirmed robustness assumptions.

### Statistical analysis

All data were performed as means ± standard error of mean (SEM). Statistical analysis were performed using GraphPad Prism 8.0.1 with the Tukey’s *t*-test for two groups and one-way ANOVA for multiple groups. The values of *p*<0.05 were considered statistically significant. Statistical significance is displayed as *p < 0.05 or **p < 0.01.

## Results

### Intramuscular iron supplementation reduces the diarrhea rate and alleviates the inflammation of infected piglets

**Figure 1:**
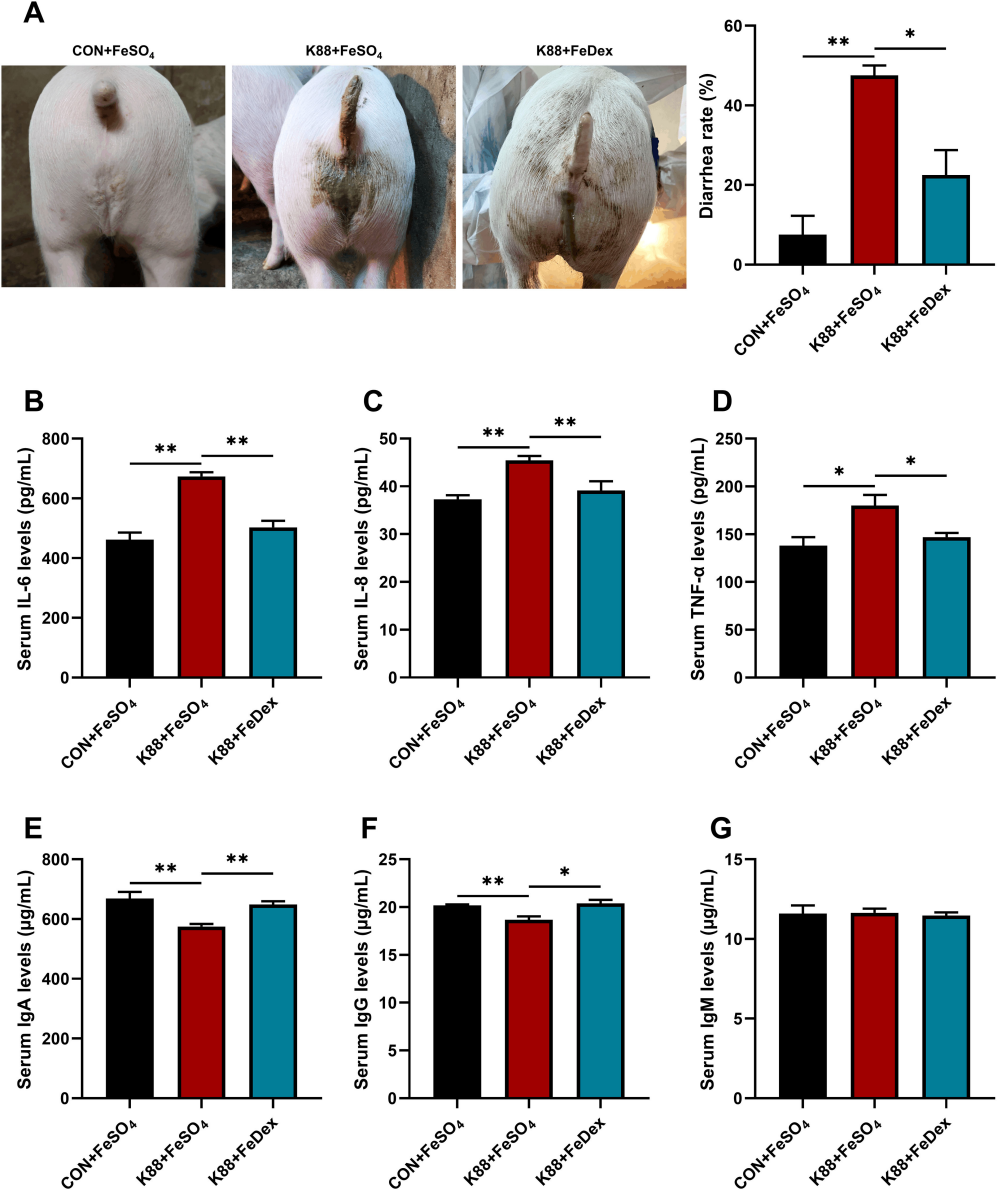
Intramuscular iron supplementation reduces the diarrhea rate and alleviates the inflammation of infected piglets. **(A)** Representative images for diarrhea, and diarrhea rate was calculated at the end of experiment. **(B-D)** Concentrations of proinflammatory cytokines including IL-6, IL-8, and TNF-α in the serum of piglets were detected by ELISA. **(E-G)** Concentrations of immunoglobulins including IgA, IgG, and IgM in the serum were measured by ELISA. n = 4 for each group. Data are expressed as means ± SEM. *P* values were calculated using Student’s *t*-test between two groups. Asterisk indicates significant difference, *P < 0.05, **P < 0.01.

Piglets challenged by ETEC K88 showed symptoms of infection such as lethargy and reduced activity. Feces from healthy piglets are dry and in clumps, whereas those from infected piglets appeared yellowish and watery (**Figure 1A**). ETEC K88 challenge markedly increased the diarrhea rate (*P<0.01*). However, compared to the K88+FeSO_4_ group, the diarrhea rate in the K88+FeDex group was decreased by 52.6% (*P<0.05*) (**Figure 1A**). Meanwhile, ETEC K88 challenge significantly (*P<0.05*) elevated the concentrations of pro-inflammatory cytokines including interleukin (IL)-6, IL-8, and tumor necrosis factor (TNF)-α in the serum of piglets (**Figures 1B–D**). Compared with K88+FeSO_4_ group, the levels of IL-6, IL-8 and TNF-α in the serum of K88+FeDex group were decreased by 25.4% (*P<0.01*), 13.9% (*P<0.01*) and 18.4% (*P<0.05*) (**Figures 1B–D**). Moreover, compared to the CON+FeSO_4_ group, piglets in the K88+FeSO_4_ group exhibited a significant (*P<0.01*) decrease of serum IgA and IgG levels (**Figures 1E–G**), indicating a decline in the resistance of piglets with diarrhea. On the contrary, the levels of IgA and IgG in the serum of K88+FeDex group were significantly (*P<0.05*) higher than those in the K88+FeSO_4_ group (**Figures 1E–G**). These findings suggested that intramuscular iron supplementation can effectively reduce the diarrhea, decrease the secretion of pro-inflammatory cytokines, and increase the levels of immunoglobulins in serum of infected piglets when compared to oral iron supplementation.

### Intramuscular iron supplementation attenuates intestinal villi injury of infected piglets

**Figure 2:**
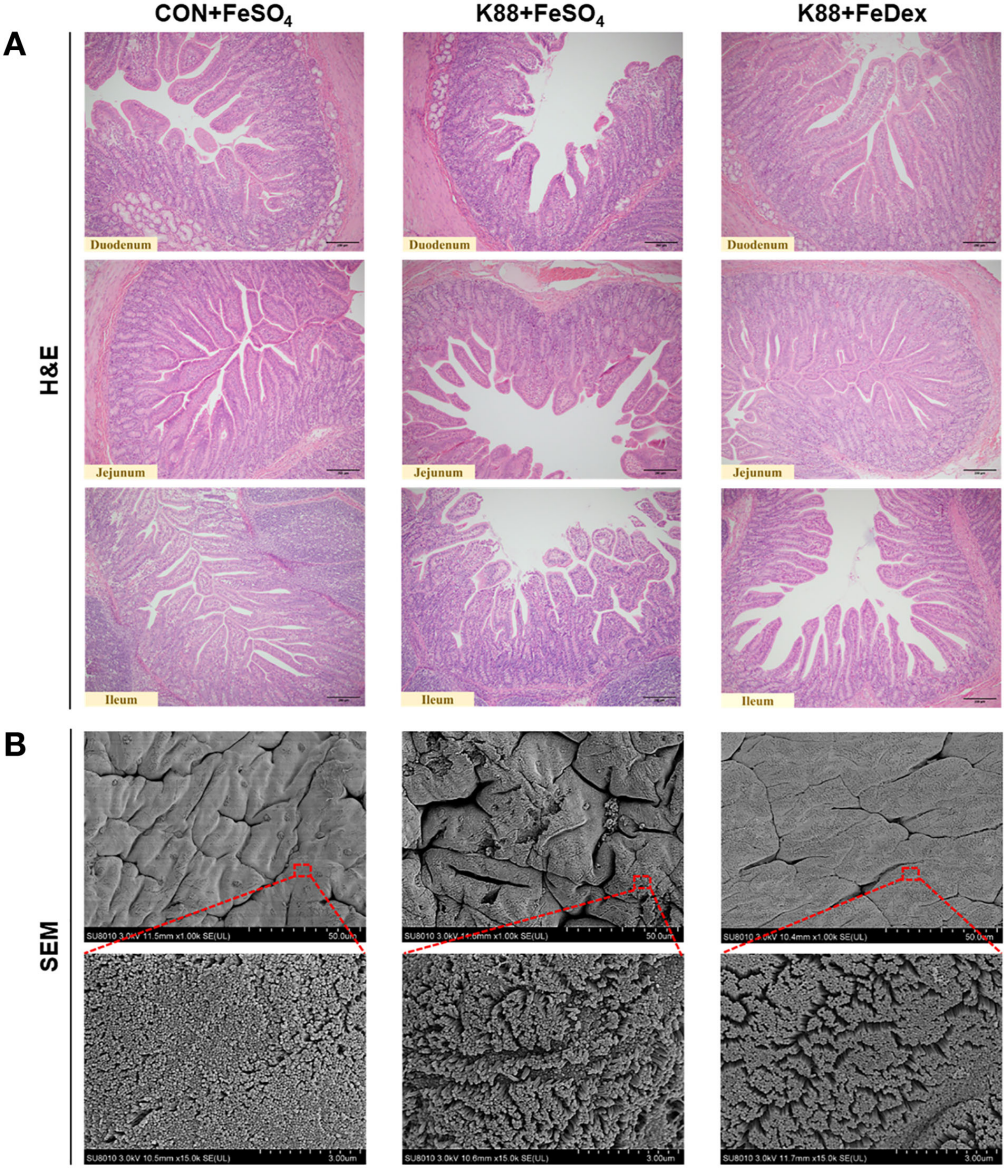
Intramuscular iron supplementation attenuates intestinal villi injury of infected piglets. **(A)** Representative images for epithelial morphology by H&E staining in the duodenum, jejunum, and ileum (×200 magnification). **(B)** Representative images for duodenal microvilli by SEM analysis.

H&E staining showed that ETEC K88 challenge exhibited noticeable villus atrophy and shedding in the duodenum, jejunum, and ileum, indicating intestinal structural damage (**Figure 2**). However, compared to K88+FeSO_4_ group, K88+FeDex group showed more organized and orderly small intestine villi and evident repair of villus tip injuries (**Figure 2**), which indicated that intramuscular iron supplementation significantly improved the intestinal morphology of infected piglets. Moreover, SEM analysis of the duodenum revealed irregular microvilli in the K88+FeSO_4_ group, whereas intramuscular iron supplementation markedly improved the microvilli structure in bacterial infected piglets (**Figure 2**). These findings suggested that intramuscular iron supplementation facilitates faster restoration of intestinal morphological damage caused by ETEC K88 infection compared to oral iron supplementation.

### Intramuscular iron supplementation enhances intestinal tight junctions of infected piglets

**Figure 3:**
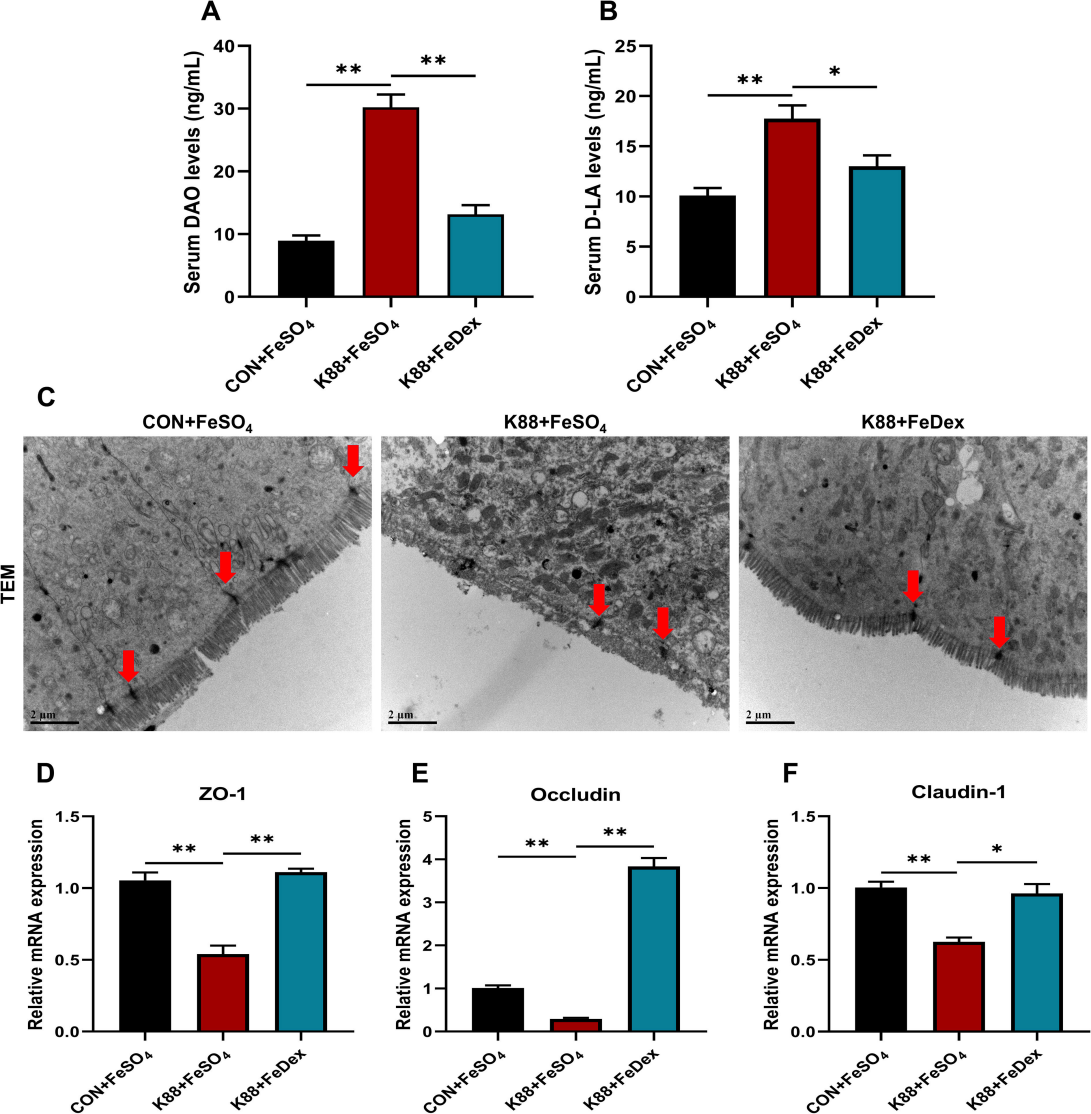
Intramuscular iron supplementation enhances intestinal tight junctions of infected piglets. **(A, B)** Serum levels of DAO and D-LA. **(C)** Tight junction (TJ) structure of duodenal epithelium were determined by TEM (×10,000 magnification), the intervals (red arrowheads) between the intestinal epithelial cells were indicated. **(D-F)** The mRNA expression levels of TJ protein genes including *ZO-1*, *Occludin*, and *Claudin-1* in the duodenum. n = 4 for each group. Data are expressed as means ± SEM. *P* values were calculated using Student’s *t*-test between two groups. Asterisk indicates significant difference, *P < 0.05, **P < 0.01.

Intestinal epithelial barrier is not only an important place to absorb nutrients, but also a key part to prevent the invasion of bacteria and macromolecules. Diamine oxidase (DAO) is a highly active intracellular enzyme found in the villi of the small intestinal mucosa, serving as an indicator of the integrity and extent of damage to the intestinal mechanical barrier. D-lactic acid (D-LA) is a product of fermentation by various intestinal microbiota. Under normal circumstances, serum levels of DAO and D-LA are relatively low. However, when there is impairment in intestinal barrier function, the levels of DAO and D-LA increase significantly. Our data showed that serum levels of DAO and D-LA were significantly (*P<0.01*) increased in piglets infected with ETEC K88 compared to the control group (**Figures 3A, B**). However, compared with K88+FeSO_4_ group, the serum levels of DAO and D-LA in K88+FeDex group were decreased by 56.5% (*P<0.01*) and 26.3% (*P<0.05*), respectively (**Figures 3A, B**). It suggested that intramuscular iron supplementation, as opposed to oral supplementation, could effectively reduce intestinal permeability in piglets experiencing diarrhea. In addition, TEM analysis revealed that the microstructure of intestinal epithelial cell microvilli were messy and broken after challenge, while K88+FeDex group showed more well-aligned and orderly, which indicated that intramuscular iron supplementation could restore the damage of microvilli (**Figure 3C**). Though no significant changes were observed in the tight junctions (TJ) structure of three groups under TEM, the mRNA levels of intestinal TJ protein-related genes including zonula occludens-1 (*ZO-1)*, *Claudin-1* and *Occludin* were all significantly (*P<0.01*) down-regulated in K88+FeSO_4_ group when compared with CON+FeSO_4_ group (**Figures 3D–F**). On the country, the transcripts of *ZO-1*, *Claudin-1* and *Occludin* in K88+FeDex group were 2.1-fold (*P<0.01*), 1.5-fold (*P<0.05*), and 13.2-fold (*P<0.01*) higher than those of K88+FeSO_4_ group. These results indicated that intramuscular iron supplementation could significantly restore the microstructure of intestinal epithelial cell microvilli and upregulate the expression of TJ protein-related genes in infected piglets compared to oral iron supplementation.

### Intramuscular iron supplementation reduces intestinal immune cell infiltration and inflammation of infected piglets

**Figure 4:**
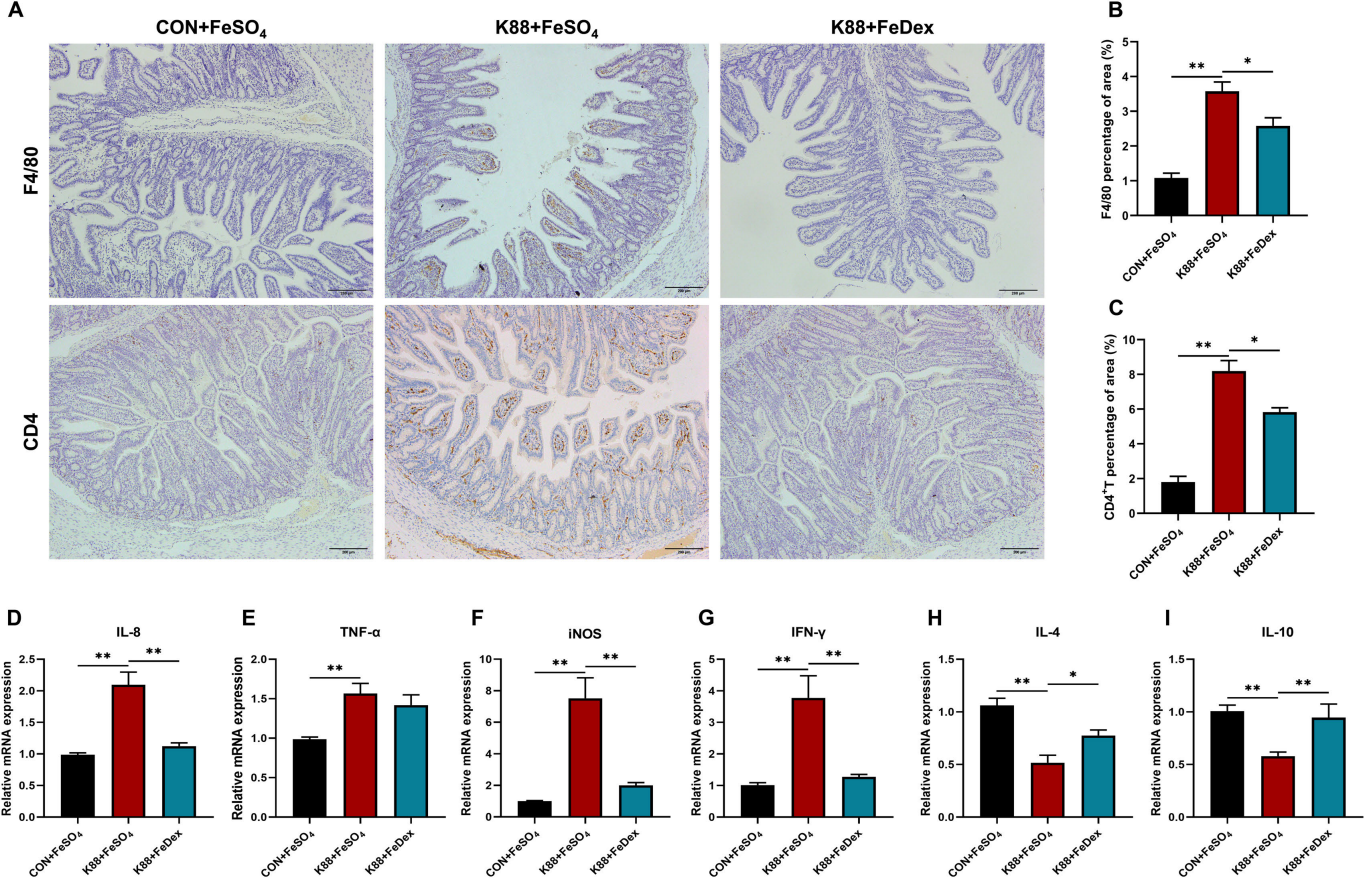
Intramuscular iron supplementation reduces intestinal immune cell infiltration and inflammation of infected piglets. **(A)** Representative images of the infiltration of F4/80 microphages in the ileum and CD4^+^ T cells in the jejunum. **(B, C)** Quantification of positive F4/80 microphages and CD4^+^ T cells in five random fields of view/each section using the Image J software. **(D-I)** The mRNA expressions of *IL-8, TNF-α, iNOS, IFN-γ, IL-4* and *IL-10* were determined by RT-PCR in the jejunum. n = 4 for each group. Data are expressed as means ± SEM. *P* values were calculated using Student’s *t*-test between two groups. Asterisk indicates significant difference, *P < 0.05, **P < 0.01.

Immunohistochemical analysis demonstrated that ETEC K88 challenge increased the infiltration of ileal microphages by 2.3-fold (*P<0.01*) and jejunal CD4^+^ T cells by 6.6-fold (*P<0.01*) of piglets (**Figures 4A–C**). However, when compared to the K88+FeSO_4_ group, the infiltration of F4/80 cells and CD4^+^ T cells of the K88+FeDex group decreased significantly (*P<0.05*) by 27.8% and 28.9%, respectively (**Figures 4A–C**). These findings suggest that intramuscular iron supplementation can effectively reduce the inflammatory infiltration of F4/80 macrophages and CD4^+^ T cells in the intestine of infected piglets. Additionally, IL-8, TNF-α, inducible nitric oxide synthase (iNOS) and interferon (IFN)-γ are important proinflammatory mediators in the gut during the development of intestinal inflammation. As expected, the mRNA levels of IL-1β, TNF-α, iNOS, and IFN-γ were all significantly (*P<0.01*) increased in the jejunum of piglets when challenged with ETEC K88 upon oral iron supplementation (**Figures 4D–G**), while those of anti-inflammatory factors such as IL-4 and IL-10 were markedly decreased (**Figures 4H, I**). When compared to the K88+FeSO_4_ group, the transcripts of pro-inflammatory factors including IL-8, iNOS, and IFN-γ in the jejunum of piglets from the K88+FeDex group were significantly (*P<0.01*) decreased (**Figures 4D–G**), and those of IL-4 (*P<0.05*) and IL-10 (*P<0.01*) were markedly elevated (**Figures 4H, I**). The results suggested that compared with oral iron supplementation, intramuscular iron supplementation significantly considerably decreased the expression of pro-inflammatory factors and raised the expression of anti-inflammatory factors in the jejunum of infected piglets.

### Intramuscular iron supplementation protects the number and function of intestinal goblet cells of infected piglets

**Figure 5:**
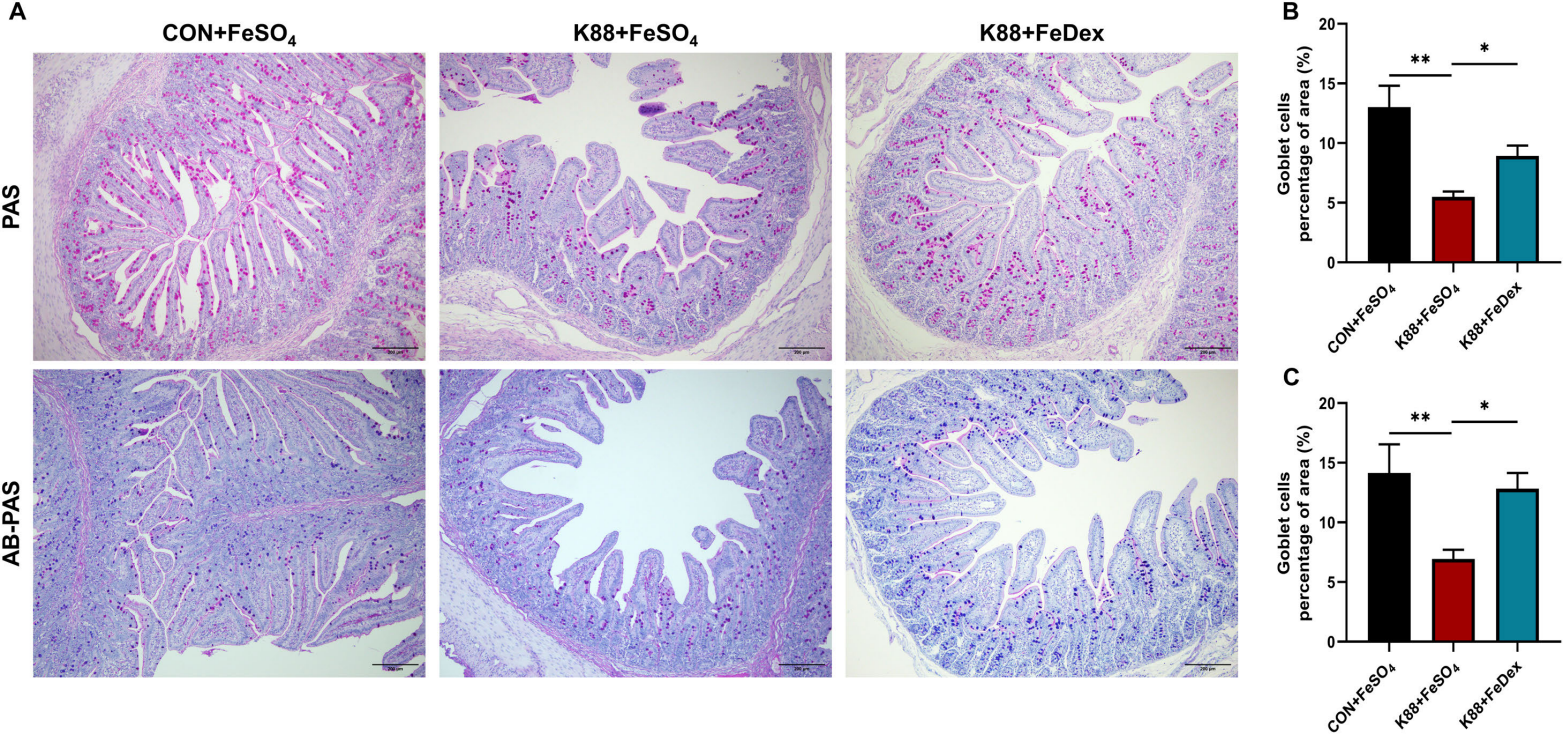
Intramuscular iron supplementation protects intestinal goblet cells of infected piglets. **(A)** Representative images of goblet cells in the jejunum (up: PAS, down: AB-PAS). Purple: neutral mucin, Light blue: acidic mucin. **(B, C)** Quantitative analysis of goblet cells. n = 4 for each group. Data are expressed as means ± SEM. *P* values were calculated using Student’s *t*-test between two groups. Asterisk indicates significant difference, *P < 0.05, **P < 0.01.

Both PAS and AB-PAS staining analysis showed that ETEC K88 infection reduced the number of goblet cells in the jejunum of piglets, which were ameliorated by intramuscular iron supplementation (**Figure 5A**). The jejunal mucin content of K88+FeDex group was 1.6-fold (PAS staining, *P<0.05*) and 1.9-flod (AB-PAS staining, *P<0.05*) higher than that of K88+FeSO_4_ group (**Figures 5B, C**). These results suggest that intramuscular iron supplementation can notably elevate mucin content in the intestines of piglets with diarrhea, thereby ameliorating goblet cell dysfunction induced by ETEC K88 and enhancing the chemical barrier in piglets affected by diarrhea.

### Intramuscular iron supplementation did not affect gut microbial homeostasis of infected piglets

**Figure 6:**
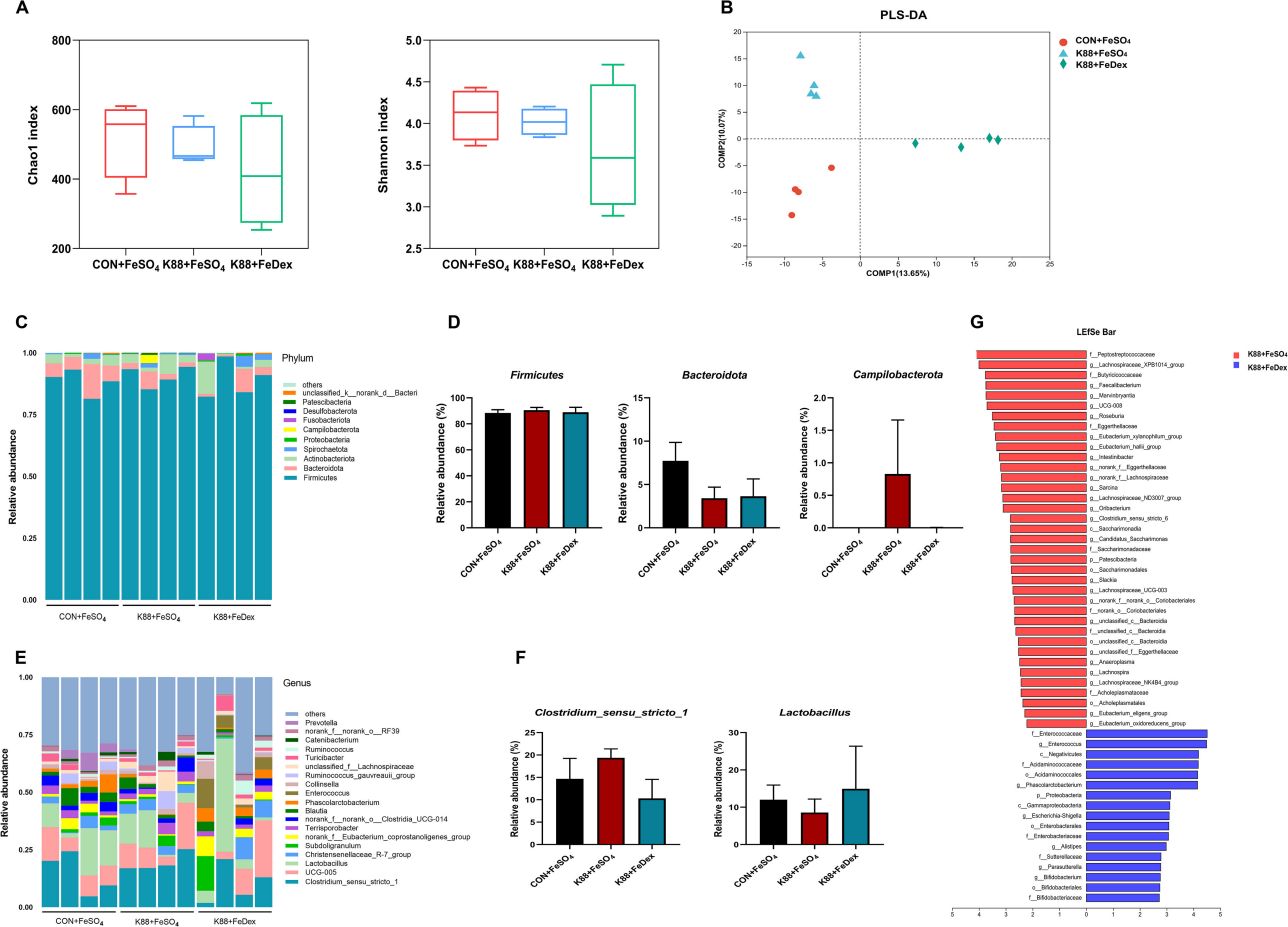
Intramuscular iron supplementation did not affect gut microbial homeostasis of infected piglets. **(A, B)** Richness and evenness measured with Chao1 and Shannon, respectively. **(C, D)** Microbiota distribution at the phylum level and relative abundance of dominated phylum. **(E, F)** Microbiota distribution at the genus level and relative abundance of dominated genus. **(G)** LEfSe analysis between K88+FeSO_4_ group and K88+FeDex group. The first letter represents the bacterial classification. c, class; o, order; f, family; g, genus. n = 4 for each group. Data are expressed as means ± SEM. *P* values were calculated using Student’s *t*-test between two groups. Asterisk indicates significant difference, *P < 0.05, **P < 0.01.

The interaction between gut microbes and the intestinal immune system is essential for maintaining mucosal homeostasis. High-throughput 16S rDNA sequencing results of cecum contents showed that there were no significant differences in Chao1 and Shannon between CON+FeSO_4_, K88+FeSO_4_, and K88+FeDex groups (**Figure 6A**), which indicated that different iron treatments had no significant effect on gut microbial species richness of piglets. Partial least squares discriminant analysis (PLS-DA) showed that each group formed different clusters, and the sample grouping effect was good, and the difference between groups was obvious (**Figure 6B**). The phylum of *Firmicutes* and *Bacteroidetes* were dominant in the cecal microbiota of CON+FeSO_4_, K88+ FeSO_4_, and K88+FeDex groups, but there were no significant differences among them (**Figures 6C, D**). The genus *Clostridium_sensu_stricto_1*, *Lactobacillus* and *Christensenellaceae_R-7_group* were the most prevalent taxa in all groups (**Figure 6E**). Compared with the K88+ FeSO_4_ group, the intestinal abundance of *Clostridium_sensu_stricto_1* was decreased and *Lactobacillus* was increased in K88+FeDex group, but the difference was not significant (**Figure 6F**). LEfSe analysis revealed 37 OTUs as significantly affected in the K88+FeSO_4_ group, and 17 OTUs in the K88+FeDex group is no significant effect on changing the composition of the dominant microbial community (**Figure 6G**). The above results showed that intramuscular iron supplementation had a certain change in the structure of intestinal flora of piglets compared with oral iron supplementation, but had no significant effect on the composition of dominant flora, and the recovery effect of intramuscular iron supplementation on intestinal flora of infected piglets was limited.

### Intramuscular iron supplementation improves iron metabolism of infected piglets

**Figure 7:**
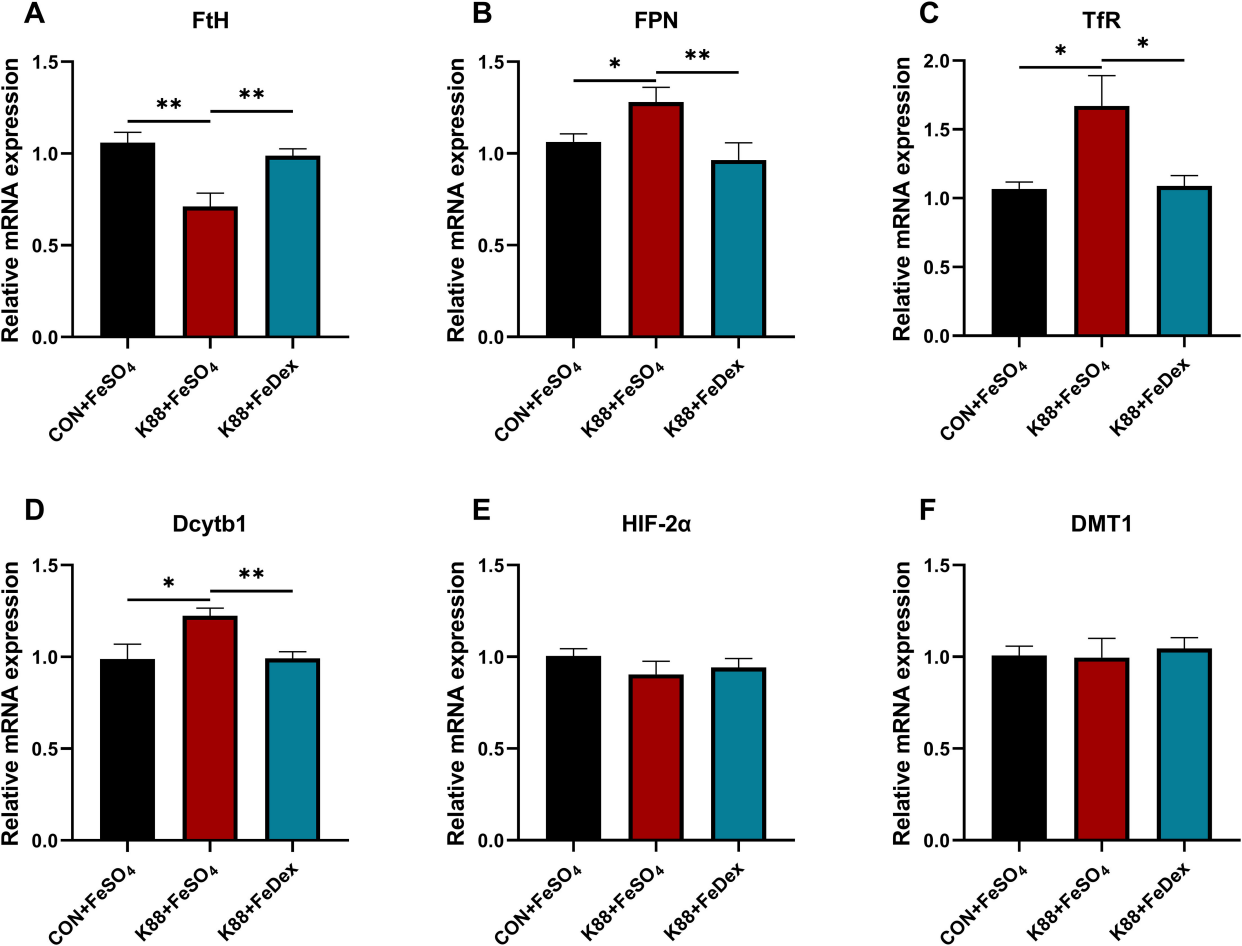
Intramuscular iron supplementation improves iron metabolism of infected piglets. **(A, F)** The mRNA expressions of *FtH*, *FPN*, *TfR*, *Dcytb1*, *HIF-2α* and *DMT1* were determined by RT-PCR in the duodenum. n = 4 for each group. Data are expressed as means ± SEM. *P* values were calculated using Student’s *t*-test between two groups. Asterisk indicates significant difference, *P < 0.05, **P < 0.01.

In order to investigate the effects of different iron supplementation methods on iron metabolism in diarrhea piglets, the levels of serum iron, unsaturated iron binding capacity (UIBC) and total iron binding capacity (TIBC) in the serum of piglets were detected. The serum iron concentration of piglets in K88+FeDex group was 1.2-fold higher (*P<0.05*) than that in K88+FeSO_4_ group, but there was no significant (*P>0.05*) change in UBIC and TIBC (**Table 2**). Compared with K88+FeSO_4_ group, the gene expression of ferritin heavy chain (*FtH*) in the duodenum of K88+FeDex group were significantly (*P<0.01*) up-regulated (**Figure 7A**), while the gene expressions of ferroportin 1 (*FPN*) (*P<0.01*), transferrin receptor (*TfR*) (*P<0.05*) and divalent metal transporter 1 (*Dcytb1*) (*P<0.01*) were significantly down-regulated (**Figures 7B–D**). The mRNA levels of hypoxia inducible factor 2 alpha (*HIF-2α*) and divalent metal transporter 1 (*DMT1*) showed no difference among groups (**Figures 7E–F**). These results indicated that iron deficiency symptoms of infected piglets were alleviated by intramuscular iron supplementation compared with oral iron supplementation.

**Table 2:** Iron status indexes in the serum.

Item (μmol/L)	Treatment			SEM	*P*
	CON+FeSO_4_	K88+FeSO_4_	K88+FeDex		
Serum iron	5.25^a^	3.95^b^	4.91^a^	0.55	<0.05
Unsaturated iron binding capacity	104.23^b^	143.53^a^	131.51^a^	16.44	<0.01
Total iron binding capacity	108.97^b^	147.48^a^	135.91^a^	16.13	<0.01

In the same row, values with no letter or the same letter superscripts had no significant diﬀerence (*P > 0.05), while values with diﬀerent letter superscripts had a significant diﬀerence (*P < 0.05). n = 4 per treatment.

## Discussion

This study demonstrated that, given the same bacterial challenge and iron supplementation, intramuscular and oral treatment developed very different intestinal phenotypic manifestations. Compared with oral iron supplementation, intramuscular iron supplementation could significantly reduce the diarrhea rate, mitigate the intestinal damage, alleviate the inflammatory response and restore the immune homeostasis of ETEC K88-infected piglets. And these beneficial effects were not due to the changes of gut microbiota, but attributed to improved iron metabolism. Recent mechanistic insights suggest that intramuscular iron administration creates systemic iron repletion while limiting luminal iron availability—a defense mechanism known as “nutritional immunity,” which restricts pathogen access to iron (Nairz et al., 2018). By elevating transferrin saturation, as shown by a higher serum iron-binding capacity in K88+FeDex compared to K88+FeSO4, this approach likely limits ETEC K88’s access to free iron. This restriction impairs the bacterium’s F4 fimbriae-mediated iron piracy mechanism (Moonens et al., 2015). Such iron sequestration may suppress bacterial siderophore syntheses, thereby mirroring the virulence attenuation observed in murine enteropathogen models (Liang et al., 2021).

The intestinal tract is the main organ for nutrient uptake, and it is also an important barrier against foreign harmful substances and pathogens. The intestinal barrier is composed of mechanical, chemical, immunological, and biological factors. The organic combination of the four barriers prevents the invasion of antigens, toxins and microorganisms, ensures the normal development of intestinal epithelial barrier and immune system, and maintains intestinal homeostasis and body health together (Di Tommaso et al., 2021). After weaning, piglets typically experience significant changes in intestinal morphology, with damage to the villi and impaired digestive and absorptive capacity (Montagne et al., 2007). Notably, oral iron supplementation in infected piglets led to luminal iron concentrations exceeding the physiological levels. This increase potentially fuels ETEC K88’s iron piracy mechanisms via F4 fimbriae, which are strategies bacteria use to acquire iron from the host (Sevilla et al., 2020). The present study revealed that oral iron supplementation in infected piglets led to marked atrophy, exfoliation of villi, enlarged villus spaces, and disorganized microvilli, accompanied by a significant reduction in the villus height-to-crypt depth ratio. This structural damage may involve iron-catalyzed reactive oxygen species (ROS) overproduction, which disrupts epithelial stem cell differentiation (Zhang et al., 2022). In contrast, intramuscular iron supplementation significantly ameliorated the intestinal damage induced by ETEC K88 infection. These findings underscore the superiority of intramuscular iron supplementation over oral administration in ameliorating intestinal morphology of weaned piglets experiencing infection, which would be good for nutrient absorption and intestinal development.

Intestinal permeability is one of the important indicators to evaluate the integrity of the intestinal barrier. Elevated levels of DAO and D-LA in serum always indicate impaired intestinal barrier integrity (Cai et al., 2019). TJ regulates paracellular permeability and plays a crucial role in intestinal barrier function. In animal models of intestinal inflammation, TJ structural damage leads to forced activation of immune cells and chronic inflammation (Suzuki, 2020). Iron restriction through parenteral administration may reduce bacterial protease secretion, thereby preserving TJ protein integrity (Ruiz-Perez and Nataro, 2014). ZO-1 and occludin are essential components of TJ and their impairment can disrupt the normal functioning of intestinal defense (Kuo et al., 2022). The intestine contains a significant amount of Claudin-1, which plays a vital role in maintaining the integrity of TJ (Furuse et al., 2002). In this experiment, compared with oral iron supplementation, intramuscular iron administration significantly decreased the levels of DAO and D-LA in serum of ETEC K88 infected piglets, but increased the expression of ZO-1, Claudin-1 and occludin in the jejunum and ileum, which indicating an decrease of intestinal permeability and increase of intestinal integrity.

As the largest immune organ of the body, the gut plays a vital role in the immune monitoring and defense function of normal intestinal flora and foreign pathogenic bacteria, which has more than 80% of the immune cells in the immune system (Wittkopf et al., 2014). The observed reduction in lamina propria CD4+ T cells and decrease in IL-6 mRNA levels suggest attenuated NF-kB activation. This phenomenon may be attributed to dual iron-mediated mechanisms: reduced biosynthesis of ETEC-derived lipopolysaccharide (LPS) under iron-restricted conditions, coupled with diminished iron-catalyzed ROS production that typically enhances inflammation (Ma et al., 2024). The gut has the biggest pool of macrophages in the body and is the largest body surface that interacts with the external environment. In face of the continuous need for the renewal of microbiota and epithelial cells, these macrophages are essential for maintaining mucosal homeostasis, and also an important part of protective immunity to against infection, which are highly phagocytic and have active bactericidal activity (Smythies et al., 2005). Studies have also shown that patients with inflammatory bowel disease (IBD) have a large accumulation of CD4^+^ T cells in the intestinal tract (Shale et al., 2013). The secretion of IL-6, IL-1β, TNF-α and other inflammatory factors is usually significantly up-regulated in animal models of intestinal inflammation (Neurath, 1998). IL-1β can stimulate the secretion and accumulation of CD4^+^ T cells in the colon and induce intestinal inflammation (Coccia et al., 2012). In this experiment, compared with oral iron supplementation, intramuscular iron supplementation effectively reduced the inflammatory infiltration of macrophages and CD4^+^ T cells, and transcription of several pro-inflammatory factors in the intestine of ETEC K88 infected piglets.

Goblet cells in the intestinal epithelium are primarily responsible for producing and secreting mucin proteins (Johansson and Hansson, 2016). The increase in jejunal mucin content following intramuscular supplementation may create an iron-chelating mucus layer that restricts pathogen iron acquisition (Scarano et al., 2023). The mucin granules produced by goblet cells are excreted onto the surface of the intestinal mucosa, helping protect and lubricate the mucous membrane (Yang and Yu, 2021). In this experiment, it was observed that oral iron supplementation significantly reduced mucin secretion in the jejunum of piglets in the infection group. In contrast, iron supplementation through injection led to a significant increase in mucin content compared to oral iron supplementation, thereby improving goblet cell damage caused by ETEC K88. This indicates that oral iron supplementation and intraperitoneal iron supplementation have different effects on different segments of the mouse intestine, influencing the direction of intestinal cell differentiation. However, the specific mechanisms behind these effects remain unclear.

Since iron is a critical component for both host and microbial life, iron supplementation can be a double-edged sword for the host during bacterial infection. On the one hand, iron supplementation improves body’s iron status and boost host’s immunity. This study revealed that intramuscular administration elevated serum iron in the oral administration group. This increase enhanced the macrophage’s bactericidal capacity, which is beneficial for immune response, thereby avoiding luminal iron overload (Nairz et al, 2018). On the other hand, iron supplementation can promote the proliferation of intestinal harmful bacteria and increase their infectivity. Studies have shown that both intravenous and oral iron therapies can impact the balance of gut microbiota in patients with IBD, although the effects on bacterial species and fecal metabolites may differ (Werner et al., 2011; Lee et al., 2017). However, our present study showed that iron supplementation had little effect on the microbial diversity of ETEC K88 infected piglets. It is understandable that intramuscular iron supplementation does not directly interact with the gut microbiota, as it bypasses the gastrointestinal tract. But it is a little puzzling that oral iron supplementation even infection didn’t change the microbiota. This may be due to the fact that the infective dose was too low to maintain the infection. Another possible reason is that the current design had insufficient sensitivity to detect moderate effect sizes, necessitating caution in interpreting negative findings.

Iron homeostasis is important for maintaining animals’ health and plays a particularly curial role in mediating host-pathogen interactions. It has long been recognized that the process of preventing bacteria from acquiring iron is crucial to host defensive mechanism (Cassat and Skaar, 2013), and infected piglets often show symptoms of iron deficiency (Guo et al., 2017). In this study, we found that ETEC K88 infection decreased the serum iron, which supposed to restrict iron available to bacterial. And intramuscular iron supplementation successfully rescued the iron deficiency symptom by an increase of serum iron. Furthermore, the expression of Dcytb1, FPN and TfR in duodenum of infected piglets with oral iron supplementation was significantly increased, while the expression of FtH was significantly decreased, which was consistent with the phenotype of iron deficiency. And intramuscular iron supplementation plays opposite role to iron metabolism with decreased expression of Dcytb1, FPN and TfR genes, and increased expression of FtH gene. These results indicated that intramuscular iron supplementation can improve iron homeostasis of infected piglets more efficiently than oral supplementation.

When providing exogenous iron to piglets, we must consider both the economic aspect of the procedure and the welfare of the animals. Piglets are prone to vomiting when fed with iron dextran because of its bad palatability. Iron dextran is a hydrophilic colloid that dissolves in water and is readily absorbed by the intestines, whose pharmacological impact is identical to that of ferrous sulfate. So in current pig farms, piglets are usually given iron supplements by intramuscular injection with iron dextran at the age of 3-5 days (Szudzik et al., 2018), and fed with ferrous sulfate in the diet after weaning. In order to fit the actual production of pigs, ferrous sulfate is used for oral iron supplement, and iron dextran is used for injection iron supplement in this study. Although intramuscular iron supplementation has been shown to be effective, its practical application is hindered by labor issues on farms. However, our study reveals the advantages of injectable iron supplementation over oral iron supplementation in ETEC K88 infected piglets, which contribute to the healthy growth of piglets and increase profitability in the farming industry. Therefore, from an overall profit perspective, utilizing FeDex for iron supplementation in managing piglets with colibacillosis and diarrhea is feasible. Our power-validated analysis (**Supplementary Table 1**) revealed high reliability for core intestinal barrier parameters (Occludin/ZO-1: f = 3.10-3.27) and metabolic markers (DAO/D-LA: f = 1.92-3.16, power > 0.93), confirming superior protection with intramuscular FeDex compared to oral FeSO4. Mucosal defense markers (PAS/AB-PAS: f = 1.12-1.45) exhibited robust detectability, whereas microbiota diversity (Chao1/Shannon: f = 0.37-0.45, power = 0.17-0.20) and specific immune components (IgM/TNF-α: f = 0.12-0.25, power = 0.05-0.41) showed limited sensitivity. These statistically grounded findings support the use of intramuscular FeDex despite higher operational costs. Future trials should refine dosing protocols using targeted parameters (f > 1.0, n≥6/group) to optimize efficacy while ensuring practical farm implementation. In future research, it would be beneficial to investigate the optimal dosage of injectable iron supplementation or compare it with other oral iron supplements to identify the most effective approach.

## Conclusion

**Figure 8:**
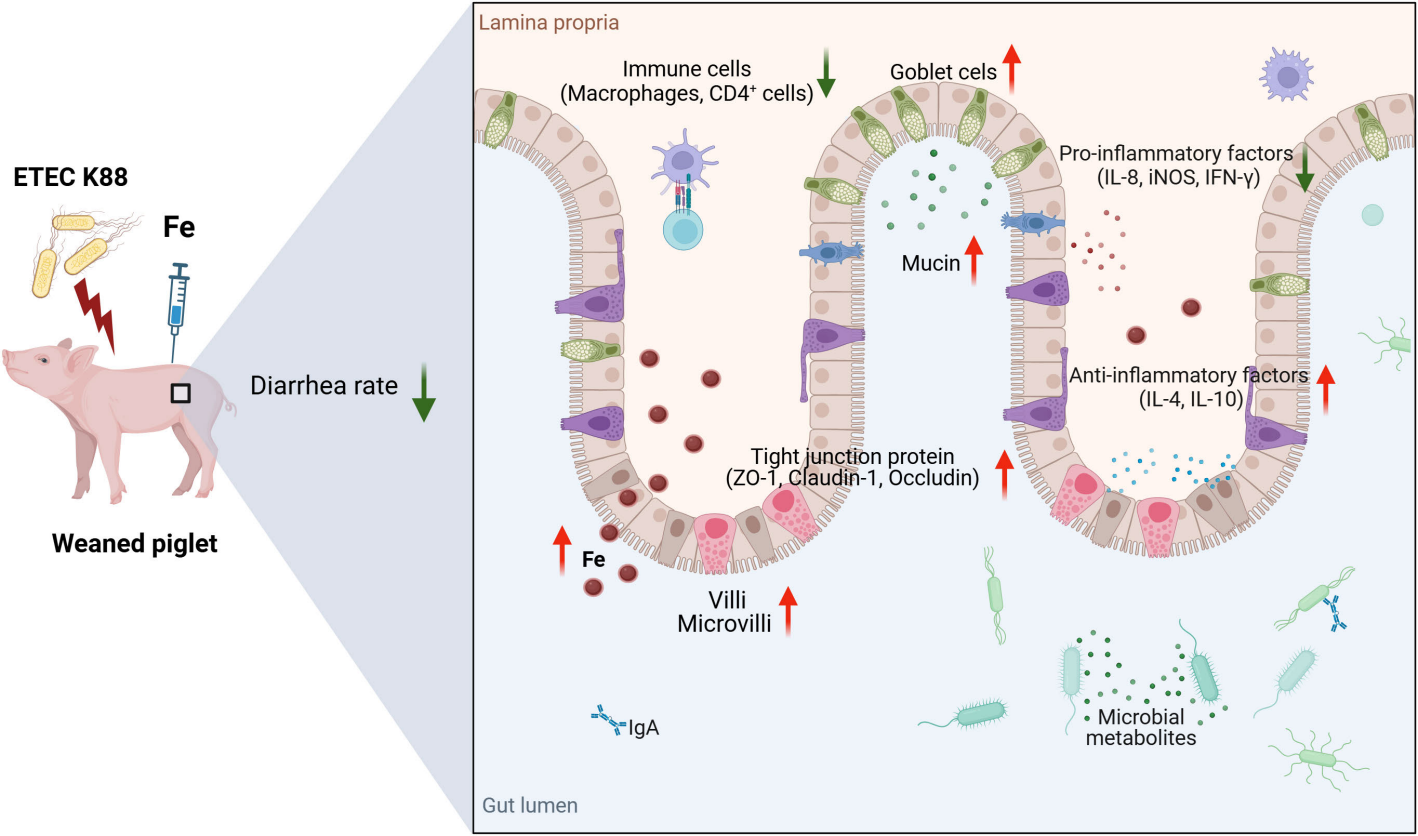
Schematic overview of the putative mechanism. Intramuscular iron supplementation improves intestinal barrier function of ETEC K88-infected piglets. The schemata was created with Biorender.com.

Intramuscular iron supplementation is beneficial to bacterial infected piglets, which improves intestinal barrier function by upregulating tight junction protein expressions, reestablishing immunological homeostasis, boosting goblet cell mucous secretion, and enhancing iron metabolism (**Figure 8**). Our finding clearly demonstrated that injectable iron supplementation is more effective during infection than oral supplementation.

## Data Availability

The original contributions presented in the study are included in the article/**Supplementary Material**. Further inquiries can be directed to the corresponding author.
