# Supplementary material for: Intramuscular iron supplementation enhances intestinal barrier function in weaned piglets challenged with enterotoxigenic Escherichia coli
Source: Front Cell Infect Microbiol. 2025 Jul 17;15:1553639. doi: 10.3389/fcimb.2025.1553639 (PMC12310581; doi:10.3389/fcimb.2025.1553639)
Supplement: Supplementary file 1 [file Table1.docx]

| **Parameter Type** | **Indicator** | **f** | **Power** |
| --- | --- | --- | --- |
| Microbiome Diversity | Chao1 Index | 0.403 | 0.17 |
|  | Shannon Index | 0.45 | 0.20 |
| Mechanical Barrier | Occludin | 3.10 | >0.999 |
|  | ZO-1 | 3.18 | >0.999 |
|  | Claudin-1 | 3.27 | >0.999 |
| Chemical Barrier | PAS Staining | 1.12 | 0.99 |
|  | AB-PAS Staining | 1.45 | >0.999 |
| Immune Barrier | Secretory IgA | 1.557 | 0.98 |
|  | IgG | 1.46 | 0.97 |
|  | IgM | 0.112 | 0.05 |
|  | CD4+ T cells | 3.01 | >0.999 |
|  | F4/80+ Macrophages | 1.89 | 0.99 |
| Biological Barrier | Diarrhoea Rate | 0.96 | 0.70 |
| Inflammation | IL-6 | 2.62 | 0.99 |
|  | TNF-α | 0.951 | 0.69 |
|  | iNOS | 4.03 | >0.999 |
|  | IL-4 | 1.73 | 0.99 |
|  | IFN-γ | 6.245 | >0.999 |
| Metabolic Markers | DAO | 1.92 | 0.99 |
|  | D-LA | 3.16 | >0.999 |

**Supplementary Table1：**Post hoc power analysis of intestinal barrier outcomes in ETEC-challenged piglets receiving intramuscular iron supplementation

Power analysis revealed profound and reliable effects (f >3, Power >99.9%) for tight junction proteins (Occludin, ZO-1, Claudin-1) and pro-inflammatory mediators (iNOS, IFN-γ), validating iron overload’s disruptive impact on intestinal integrity. In contrast, microbial diversity indices (Chao1/Shannon) and IgM exhibited minimal responsiveness (f <0.5, Power <20%), suggesting either biological insensitivity or insufficient sample size. Moderate effects (Power 70–99%) for diarrhoea rate and TNF-αnecessitate expanded validation. Collectively, these data prioritize high-impact targets (e.g., mechanical barrier proteins) for therapeutic translation."
